# On Man's Power over Himself to Prevent or Control Insanity

**Published:** 1844-01

**Authors:** 


ART. V.—*On Man's Power over himself to prevent or control Insanity.* By the Rev. JOHN BARLOW, M.A. F.R.S. &c., Secretary of the Royal Institution of Great Britain.—*London*, 1843. 18mo, pp. 68.

THIS little essay, like the former one from the same pen which we noticed with approbation a few months since, contains the substance of a communication made to the members of the Royal Institution at one of their Friday evening meetings. It possesses the same claims to the attention of the general reader, in the truthfulness, simplicity, and at the same time the comprehensiveness of its views; although the intelligent medical reader may probably not find anything in it, with which he is not already familiar. Mr. Barlow's object is to prove that, in a large proportion of cases, insanity may be traced to the faulty indulgence of some propensity or feeling, which the due exercise of self-control would have restrained; and he justly appeals, in support of this position, to the valuable effects resulting from the application of this principle to the treatment of insanity—the best restraint under which lunatics can be kept being that of *their own* self-control, if motives can be found of sufficient strength to cause them to exert it.

The views entertained, and the principles elucidated in this little work, have indeed a wide and important range. They call the attention to that greatest of all sciences which teaches us to govern and to strengthen the highest of our faculties for the most valuable ends, and to make the intellect the great auxiliary of virtue. It is gratifying to find an accomplished clergyman addressing a highly-cultivated audience on these topics; worthy of those addressed, however distinguished in philosophy,—and worthy of a divine, who should know how to address himself to minds of the highest as well as of the humblest attainments. From the pages of Mr. Barlow's essay powerful arguments may be gathered for the promotion of education, and of all other means of preventing criminal excesses, and warding off impulses that by repetition become morbid and incontrollable; and in these days of rapid movement, vast speculation, and growing avarice and ambition, there are many readers in many classes of society to whom a medical practitioner may recommend such reading as remedial against restless cares which "not poppy, nor mandragora, nor all the drowsy syrups in the world" can cure.

